# Simultaneously Cooperative, but Serially Antagonistic: A Neuropsychological Study of Diagonistic Dyspraxia in a Case of Marchiafava-Bignami Disease

**DOI:** 10.1155/2008/845341

**Published:** 2008-07-15

**Authors:** Kazumi Hirayama, Kaori Tachibana, Nobuhito Abe, Hideaki Manabe, Takahisa Fuse, Tetsuro Tsukamoto

**Affiliations:** ^1^Department of Behavioral Neurology and Cognitive NeuroscienceTohoku University Graduate School of MedicineSendaiJapan; ^2^Department of NeurosurgeryNational Hospital OrganizationShizuoka Medical CenterShizuokaJapan; ^3^Department of Cardiovascular SurgeryNational Hospital OrganizationShizuoka Medical CenterShizuokaJapan; ^4^Department of NeurologyNumazu Rehabilitation HospitalNumazuJapan

**Keywords:** Corpus callosum, diagonistic dyspraxia, Marchiafava-Bignami disease

## Abstract

We describe a patient with Marchiafava-Bignami disease who showed, in addition to signs of callosal interruption, a peculiar form of diagonistic dyspraxia. Unlike the typical diagonistic dyspraxia, both of the patient’s hands could simultaneously cooperate in a sequence of bimanual actions. More specifically, his right hand could start a commanded action with the cooperation of his left hand. However, once the action was completed, his left hand started an antagonistic action, undoing the result, with the cooperation of his right hand. Once this countermanding action was completed, the original action started again. These antagonistic actions repeated themselves alternately unless he was restrained. The patient's diagonistic dyspraxia was apparent in only some bimanual actions, and he showed no diagonistic dyspraxia when performing voluntary actions; the antagonistic actions occurred in response to oral commands or by imitation. Magnetic resonance imaging showed symmetrical demyelination with partial necrosis in the genu, body, and anterior splenium of the corpus callosum. We speculate that the bimanual coordination is possible because part of the corpus callosum is intact, whereas the antagonistic actions may be caused by conflict between the two hemispheres due to interhemispheric disinhibition elicited by the demyelinated part of the corpus callosum.

## 1. Introduction

Marchiafava-Bignami disease is an extremely rare complication of chronic alcoholism characterized by the pathologic feature of symmetrical demyelination of the corpus callosum with or without necrosis [17]. This demyelination is clearly visible on magnetic resonance imaging (MRI) [7,8,13]. Due to the corpus callosum lesions, this disease can show signs of callosal interruption, including left-handed agraphia [5,6,12, 13,15], left-handed ideomotor apraxia [5,6,12,13,15], right-handed constructional disability [5,6], alexia or anomia in the left visual field [5,6,15], crossed hemispatial neglect [12], auditory extinction in the left ear [6, 12], tactual anomia in the left hand [12], and diagonistic dyspraxia [6].

ISSN 0953-4180/08/$17.00 © 2008 – IOS Press and the authors. All rights reserved

Diagonistic dyspraxia is usually defined as a transient conflict between the two hands following corpus callosum lesions. Akelaitis and colleagues first described, in the patients who had undergone callosotomy, involuntary movements of the left hand which acted in an opposite way to the actions executed by the right hand [2,3]. For example, a patient dresses with the right hand, while simultaneously undressing with the left hand. Since their reports, some researchers have described the patients with diagonistic dyspraxia and related syndromes [1,14,18,19,23,24].

In this report, we describe a patient with Marchiafava-Bignami disease who showed not only signs of callosal interruption but also a peculiar form of diagonistic dyspraxia. Unlike the typical form of diagonistic dyspraxia, when the patient was asked to perform a bimanual action, his right hand started the action and his left hand simultaneously cooperated with the right hand. However, as soon as the action was completed, his left hand started an opposite action, undoing the result of the original action, and the right hand cooperated with the left hand. Furthermore, once this countermanding action was completed, the patient started to perform the original action again. These antagonistic behaviors were repeated alternately and continued until he was restrained. We speculate that the bimanual coordination is possible because part of the corpus callosum is intact, whereas the antagonistic actions may be caused by conflict between the two hemispheres due to interhemispheric disinhibition elicited by the demyelinated part of the corpus callosum.

## 2. Case report

A 53-year-old right-handed man with a 9th-grade education, who suffered from diabetes and arteriosclerosis obliterans, underwent a femoropopliteal bypass graft operation in hospital. The patient had a long history of alcoholism, drinking more than 1000 mL of Japanese rice wine every day. Although multi-vitamins were administered intravenously during the patient's recovery from his operation, he began to show confusion, incontinence, and gait disturbance 4 days postoperatively. A cranial computed tomographic scan and MRI showed symmetrical demyelination of the corpus callosum with partial necrosis. The patient's confusion and gait disturbance disappeared within a few weeks, but his incontinence remained unchanged. At this time, he showed deterioration of intelligence and memory disturbance. MRI performed 82 days after onset showed symmetrical demyelination with partial necrosis in the genu, body, and anterior splenium of the corpus callosum. The most anterior parts of the genu and posterior splenium were preserved. An additional small lesion was evident in the medial portion of the right thalamus (Fig. 1).

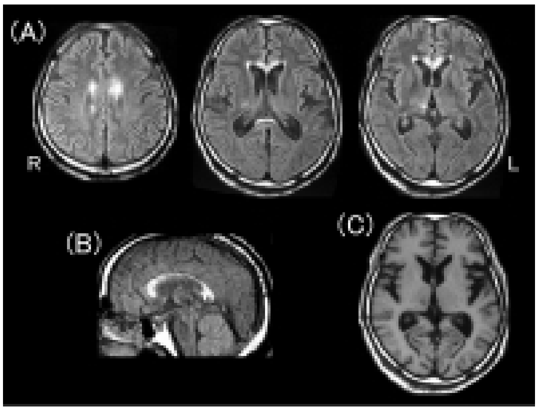

Fig. 1. Magnetic resonance imaging (MRI) performed 82 days after onset. Fluid attenuated inversion recovery (FLAIR) images in (A) axial and (B) sagittal planes revealed a symmetrical hyperintensity area in the genu, body, and anterior splenium of the corpus callosum, suggesting demyelination of these areas. The most anterior parts of the genu and posterior splenium were preserved. An additional small lesion was detected in the medial portion of the right thalamus. T1-weighted image in the axial plane (C) showed a hypointensity area in the corpus callosum, which was a part of the hyperintensity area revealed in the FLAIR images, suggesting partial necrosis of the demyelinated lesion. The hypointensity area was also evident in the right thalamus, suggesting an infarction.

The patient was transferred to our hospital 4 months after the onset. His level of consciousness was normal, and no deficit in temporal or spatial orientation was noted. His position sense and two-point discrimination on the left hand were impaired, but his sensations to pain, temperature, and vibration were normal, suggesting a sensory disturbance of cortical origin. He was totally deaf in his right ear, which was caused by a previous trauma. General neuropsychological assessments revealed that his intelligence and attention were impaired. Although he experienced no difficulty in everyday life, neuropsychological tests revealed deterioration of episodic memory. There was no evidence of aphasia or buccofacial apraxia. His executive function was not tested because of the difficulty interpreting the result due to his impaired intelligence. Table 1 summarizes the results of the general neuropsychological assessments with the available normative data [9,11,21, 22].

Among the patient's cognitive deficits, the most distinctive symptom was his peculiar form of diagonistic

Table 1
General neuropsychological findings

| Neuropsychological test | | Performance | Norms |
|---|---|---|---|
| *Intelligence* | | | |
| Wechsler Adult Intelligent Scale-Revised | Verbal IQ | 63 | |
| | Performance IQ | 45 | |
| | Full IQ | 49 | |
| Raven Coloured Progressive Matrices* | | 24 | 29.3 (6.7) |
| *Attention* | | | |
| Digit span** | Forward | 3 | 5.25 (1.29) |
| | Backward | 3 | |
| Tapping span** | Forward | 3 | 4.69 (0.82) |
| | Backward | 3 | |
| *Memory* | | | |
| Wechsler Memory Scale-Revised*** (WMS-R) | Verbal Paired Associates | 9/24 | 16.7 (3.8) |
| | Delayed Recall of Verbal Paired Associates | 5/8 | 7.0 (1.2) |
| *Language* | | | |
| Western Aphasia Battery* (WAB) | Aphasia Quotient (AQ) | 94.9 | 97.7 (3.0) |
| | Cortical Quotient (CQ) | | |
| | Right hand | 90 | 96.3 (4.4) |
| | Left hand | 84.8 | |

Norms are expressed as means, with standard deviations in parentheses.
*Norms from Sugishita, 1986 [21].
**Norms from Fujii, 2002 [9].
***Norms from Sugishita, 2001 [22].

dyspraxia, which was observed only during the neuropsychological assessments. Although this was not apparent in his everyday voluntary actions, he claimed that his left hand often did not do what he wanted it to do. In view of these clinical observations, we investigated his specific type of diagonistic dyspraxia in more depth.

The patient gave written informed consent to participate in a series of experimental investigations to determine the features of his diagonistic dyspraxia. The study was performed in accordance with the Declaration of Helsinki.

## 3. Experimental investigations

### 3.1. Callosal disconnection syndrome

We first evaluated the callosal disconnection syndrome underlying the patient's specific type of diagonistic dyspraxia. He showed agraphia (Fig. 2-A) and ideomotor apraxia of the left hand. The apraxia was worst with oral commands, but improved slightly with imitations and actual object use. Most of the errors were perseverations of a previous reaction or amorphous movements. Constructional disability was shown in both hands on Block Design in the Wechsler Adult Intelligence Scale-Revised [20], but the errors were different in terms of quality in each hand (Fig. 2-B). Examinations using a tachistoscope revealed neither alexia nor anomia in the left visual field. The patient showed no crossed hemispatial neglect, pathological grasping, or compulsive use of tools. Dichotic listening tests could not be performed due to the deafness in his right ear. Table 2 summarizes the neuropsychological findings relevant to callosal disconnection.

An intriguing phenomenon was observed during the assessments of tactual naming ability. A few seconds after we covered both of his eyes with eye patches and explained the tasks to be performed, he suddenly shouted, "I don't like it!", took off each eye patch with the corresponding hand (i.e., the left eye patch with the left hand and the right eye patch with the right hand), left the examination room, and returned to his ward, where he sat on a chair. However, when asked the reason for his dislike of the test, he looked blank and said, "I don't know the reason. I don't dislike it at all. Let's do it now." However, we eventually had to abandon any attempts at assessing his tactual naming ability.

A notable disconnection phenomenon was also observed when he performed the Visual Paired Association task in the Wechsler Memory Scale-Revised (WMS-R) [22]. On this task, the patient was shown several pairs of a figure and a color and was asked to memorize the association. He was then shown only a figure and asked to point with his right hand to the corresponding associated color. His right hand performed this task poorly. However, his left hand also pointed to the same or a different color, always after a short

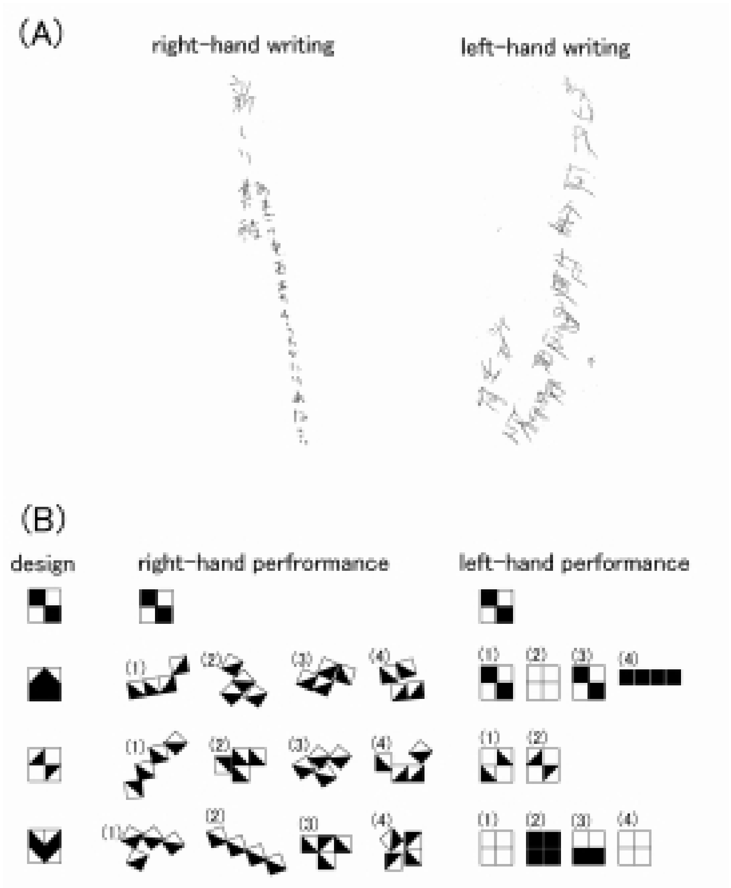

Fig. 2. (A) Examples of the writing to dictation by the patient. The sentence written by his right hand showed no abnormality except for a few errors of Kanji (ideogram) characters (due to his low level of education) and a few errors of Kana (phonogram) letters (due to mishearing). In contrast, the sentence written by his left hand consisted of incorrect letter-like items, most of which resembled real Kanji characters or incorrect combinations of Kanji parts. (B) Examples of the block design by the patient. Numbers in parentheses indicate the temporal order of his responses when he was requested to construct with the blocks the design depicted in the left column. In three out of four trials, his right hand could not reproduce the correct designs. The global shape of the design (a square) was also not constructed. His left hand failed to reproduce the correct designs, but succeeded in constructing the global shape of the design as a square.

delay. Surprisingly, the performance by his left hand was noticeably better than that of his right hand. Most of the time, the patient appeared to be unaware of the behavior of his left hand, but on the occasions when he was aware of it, he looked at his left hand and shifted it to the color at which his right hand was pointing. When he was asked to point to the correct color with his left hand, he initially did so, but on some occasions after looking at his left hand, it pointed to another color. Thus, the final performance of his left hand was similar to that of his right hand (Table 2).

### 3.2. *A peculiar form of diagonistic dyspraxia*

During his hospitalization, the patient stated that his left hand often does not do what he wants it to do, and therefore we evaluated his actions. When he was asked to manipulate tools, his hands showed a simultaneously cooperative but serially antagonistic behavior. For example, when he was asked to open the cap of a plastic bottle, his right hand picked up the bottle at the cap, brought it towards him, and opened it, while his left hand helped his right hand by holding the bottle. However, as soon as the cap was off the bottle and was put on the table, his left hand picked it up, put it on the bottle, and capped the bottle again, while his right hand helped his left hand by holding the bottle. Furthermore, when his left hand pushed the bottle towards us, his right hand followed it, picked up the bottle at the cap again, and opened it, while his left hand held the bottle (Fig. 3). During the whole sequence of the opening and closing operations, one hand always helped the other

Table 2
Neuropsychological findings relevant to callosal disconnection

| Assessment | | Performance | Norms |
|---|---|---|---|
| *Praxis* (*in WAB*) | | | |
| Upper limb | Right hand (oral command, imitation) | 10/10 (5/5, 5/5) | |
| | Left hand (oral command, imitation) | 4/10 (2/5, 2/5) | |
| Use of tool | Right hand (oral command, imitation, actual object) | 15/15 (5/5, 5/5, 5/5) | |
| | Left hand (oral command, imitation, actual object) | 6/15 (0/5, 1/5, 2/5) | |
| Facial | | 15/15 | |
| Pantomime of complex actions (with both hands) | | 0/15 | |
| *Tachistoscopic reading* | | | |
| Kana (phonogram) letters | Right visual field | 23/24 | |
| | Left visual field | 22/24 | |
| Kanji (ideogram) letters | Right visual field | 15/24 | |
| | Left visual field | 20/24 | |
| *Tachistoscopic naming* | | | |
| Objects | Right visual field | 19/24 | |
| | Left visual field | 21/24 | |
| *Behavioural Inattention Test** | | | |
| Conventional subtest | Right hand | 146/146 | 131 |
| | Left hand | 130/146*** | (Cut off) |
| *WMS-R*** | | | |
| Visual Paired Associates | Right hand (the hand to use was not specified) | 5/18 | 12.2 (4.0) |
| | Left hand (the hand to use was not specified) | 9/18 | |
| | Right hand (the hand to use was specified) | 5/18 | |
| | Left hand (the hand to use was specified) | 4/18 | |
| Delayed Recall of Visual Paired Associates | Right hand (the hand to use was not specified) | 2/6 | 5.0 (1.3) |
| | Left hand (the hand to use was not specified) | 4/6 | |

Norms are expressed as mean and standard deviation in parenthesis.
*Norms from Ishiai, 1999 [11].
**Norms from Sugishita, 2001 [22].
***In the letter cancellation task, the patient could not detect one particular letter repeatedly, irrespective of which side of the space the letter was presented.
WAB, Western Aphasia Battery; WMS-R, Wechsler Memory Scale-Revised.

hand by holding the bottle. Cap opening by the right hand and cap closing by the left hand were repeated continuously until he was restrained.

It should be noted that these antagonistic behaviors were elicited by oral commands or through imitation, and were not observed in the patient's everyday voluntary behaviors. The typical diagonistic dyspraxia occurring within an action sequence was not observed in any of his actions. Although typical apraxia was occasionally observed in the patient's left hand, the antagonistic behavior was never observed when the patient was asked to perform actions that would normally be done with only one hand (e.g., combing his hair, brushing his teeth, or eating with a spoon) or without tools (e.g., shaking his fist, scratching his head, or snapping his fingers).

To examine further whether this specific form of antagonistic behavior is common to actions using both hands, we prepared several tools requiring complex bimanual coordination. The patient was asked to perform a series of bimanual actions, and two examiners judged whether or not diagonistic dyspraxia was observed in each action. The patient's specific form of diagonistic dyspraxia was apparent when he was asked to fold a sheet of paper, pull the cap off a pen, open the lid of a box, and put something into a pouch. However, the antagonistic behavior was not apparent when he was asked to fasten a shirt button, tie a piece of string, put on a tie, hold a book and turn over the pages. The difference between the actions with and without diagonistic dyspraxia might depend on the role played by the left hand in these actions (see Discussion for details). These results are summarized in Table 3.

## 4. Discussion

We have described a patient who manifested diagonistic dyspraxia with a specific trait. Based on neurological, neuroradiological, and neuropsychological assessments, and on the patient's history of chronic alcoholism, he was diagnosed with Marchiafava-Bignami disease. Detailed tests revealed that his right hand was able to start a commanded action with the coopera-

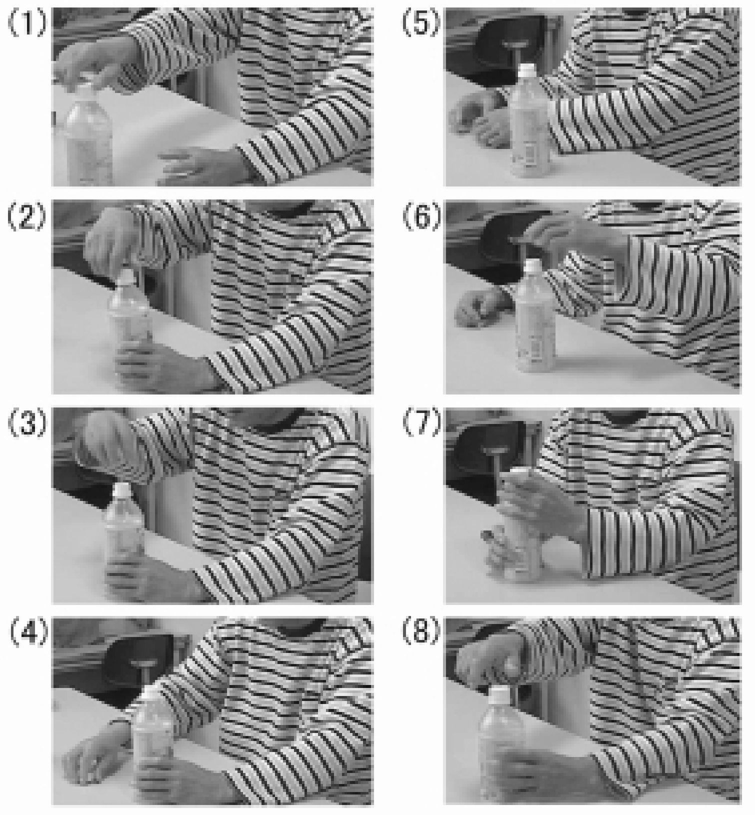

Fig. 3. An example of the diagonistic dyspraxia observed in this case. The numbers in parentheses indicate the temporal order of the patient's reactions when he was asked to open the cap of a plastic bottle. (1) His right hand grasped the bottle at the cap and brought it to him. (2) & (3) His right hand opened the cap. While his right hand was performing this action, his left hand helped his right hand by holding the bottle. (4) His right hand placed the cap on the table. (5) As soon as the cap was placed on the table, his left hand picked it up. (6) His left hand moved the cap back on to the bottle. (7) The left hand capped the bottle. While his left hand was performing this antagonistic action, his right hand helped his left hand by holding the bottle. (8) While his left hand pushed the bottle toward us, his right hand followed it to catch the bottle at the cap. Then the right hand opened the cap again, with the left hand holding the bottle. Opening by the right hand and capping by the left hand with the other hand holding the bottle were repeated until he was restrained.

tion of his left hand. However, once the action was completed, his left hand started an antagonistic action, undoing the result, with the cooperation of his right hand. Once this countermanding action was completed, the original action started again. These antagonistic actions repeated themselves alternately unless the patient was restrained. Furthermore, he showed no diagonistic dyspraxia when performing voluntarily actions, the antagonistic behaviors only being observed when he performed actions in response to oral commands or by imitation. His diagonistic dyspraxia occurred only when he performed actions requiring bimanual coordination. These simultaneously cooperative but serially antagonistic behaviors are different from those of typical forms of diagonistic dyspraxia, in which conflict occurs immediately after the start of an action (i.e., within an action sequence). In our patient, callosal interruption was caused by the demyelination of the genu, body, and anterior splenium of the corpus callosum due to Marchiafava-Bignami disease. This rare condition, along with the right thalamic infarction, might underlie the patient's diagonistic dyspraxia.

How can this specific form of diagonistic dyspraxia be accounted for? The classical theory of apraxia [16] assumes that full knowledge of an action (i.e., the name, the relationship between a tool and its action, the result and meaning of the action, etc.) is stored in the left hemisphere. In our patient, bimanual coordination may be possible because the intact part of the corpus callosum enables the left hemisphere to interact with the right hemisphere. However, the right hemisphere is likely to fail to recognize the completion of the action, as it is unable to receive the relevant information from the left hemisphere because of damage to part of the in-

Table 3
Actions with and without diagonistic dyspraxia

| Actions | Diagonistic dyspraxia |
|---|---|
| *Actions requiring one hand (in WAB Praxis)** | |
| Combing his hair | Not observed |
| Brushing his teeth | Not observed |
| Eating with a spoon | Not observed |
| Using a hammer | Not observed |
| Shutting a lock with a key | Not observed |
| Shaking his fist | Not observed |
| Making a salute | Not observed |
| Waving his hand | Not observed |
| Scratching his head | Not observed |
| Snapping his fingers | Not observed |
| *Actions requiring both hands* | |
| Opening the cap of a plastic bottle | **Observed** |
| Folding a sheet of paper | **Observed** |
| Pulling the cap off a pen | **Observed** |
| Opening the lid of a box | **Observed** |
| Putting something into a pouch | **Observed** |
| Fastening buttons on a shirt | Not observed |
| Tying a piece of string | Not observed |
| Putting on a tie | Not observed |
| Holding a book and turning over the pages | Not observed |

*Although diagonistic dyspraxia was not observed in this test, typical apraxia was observed in the patient's left hand (see Table 2 for details). WAB, Western Aphasia Battery.

terhemispheric connection. Therefore, the disconnected right hemisphere (i.e., the left hand) might initiate the antagonistic action that reverses the previous action. It should be noted that when this antagonistic action sequence was initiated, both hands were again simultaneously cooperative, possibly due to the intact part of the corpus callosum. Consistent with our interpretations, Nishikawa et al. [19] described a behavior associated with callosal disconnection, which they called "conflict of intentions," and suggested that disconnected hemispheres could have different and conflicting intentions. The phenomenon observed in Visual Paired Associates of WMS-R in our patient, in which the performance of his left hand was better than that of his right hand, also supports the idea of conflict between the hemispheres.

It should be noted that our patient's peculiar diagonistic dyspraxia was apparent in only some bimanual actions. One important difference between these two types of actions may be related to the role of the left hand (i.e., the right hemisphere) in the actions. The right hemisphere may store the motor programs for the main operation of actions that the left hand has performed repeatedly in daily life, and these motor programs may disrupt the actions as a result of interhemispheric disconnection. Consistent with this idea, the main operations of the actions showing diagonistic dyspraxia were those performed with either the left or the right hand. For example, when opening a bottle cap or folding of a sheet of paper, either the left or the right hand can perform the main operations of opening or folding, while the other hand assists it. In contrast, the main operations of the actions showing no diagonistic dyspraxia were those performed with only the right hand. For example, the right hand plays the major role in actions such as fastening a shirt button or tying a piece of string, while the left hand usually plays a subsidiary role in these actions.

Although this type of diagonistic dyspraxia has not been investigated in detail, a literature search revealed the presence of a similar symptom (along with typical diagonistic dyspraxia). For example, based on the observations by the patient's wife, Gazzaniga et al. [10] reported on a callosum-sectioned patient and described as follows; "the patient would pick up the evening paper with the right hand, but put it down abruptly with the left and then have to pick it up again with the right". In addition, Tanaka et al. [24] reported on a patient with an infarction in the corpus callosum from the genu to the most caudal part of the body and described "after he put his sock on with both hands, his left hand pulled it off". Recently, Barbeau et al. [4] reported on a patient (JJL) who had lesions of the rostrum, genu, and body of the corpus callosum. It was reported that JJL puts his shoes on, ties his laces, then unties them, takes the laces off the shoes and throws them under the bed. Expanding on these previous findings, our report further indicates that, at least in some patients, serial antagonistic actions can be performed by bimanual coordination after completion of the original actions. It also shows that this type of diagonistic dyspraxia can be characterized by factors such as the circumstances initiating the action (i.e., voluntary actions or commands/imitation), the complexity of the action (i.e., one-hand or two-hand actions), or the role of the left hand in the action.

Not all the symptoms observed in our patient were quantitatively evaluated, and there may be alternative interpretations of the pattern of his diagonistic dyspraxia. However, the present study demonstrates a specific form of diagonistic dyspraxia that sheds further light on our understanding of the actions implemented by each hemisphere. To clarify this further, detailed neuropsychological assessments of patients with more focal damage to sections of the corpus callosum, such as infarcts involving the callosal pathway, are needed.
